# Human endometrial mesenchymal stem cells exhibit intrinsic anti-tumor properties on human epithelial ovarian cancer cells

**DOI:** 10.1038/srep37019

**Published:** 2016-11-15

**Authors:** Shixia Bu, Qian Wang, Qiuwan Zhang, Junyan Sun, Biwei He, Charlie Xiang, Zhiwei Liu, Dongmei Lai

**Affiliations:** 1 The International Peace Maternity and Child Health Hospital, School of Medicine, Shanghai Jiaotong University, Shanghai, 200030, China; 2 State Key Laboratory for Diagnosis and Treatment of Infectious Diseases, the First Affiliated Hospital, School of Medicine, Zhejiang University, Hangzhou, 310003, China

## Abstract

Epithelial ovarian cancer (EOC) is the most lethal tumor of all gynecologic tumors. There is no curative therapy for EOC thus far. The tumor-homing ability of adult mesenchymal stem cells (MSCs) provide the promising potential to use them as vehicles to transport therapeutic agents to the site of tumor. Meanwhile, studies have showed the intrinsic anti-tumor properties of MSCs against various kinds of cancer, including epithelial ovarian cancer. Human endometrial mesenchymal stem cells (EnSCs) derived from menstrual blood are a novel source for adult MSCs and exert restorative function in some diseases. Whether EnSCs endow innate anti-tumor properties on EOC cells has never been reported. By using tumor-bearing animal model and *ex vivo* experiments, we found that EnSCs attenuated tumor growth by inducing cell cycle arrest, promoting apoptosis, disturbing mitochondria membrane potential and decreasing pro-angiogenic ability in EOC cells *in vitro* and/or *in vivo*. Furthermore, EnSCs decreased AKT phosphorylation and promoted nuclear translocation of Forkhead box O-3a (FoxO3a) in EOC cells. Collectively, our findings elucidated the potential intrinsic anti-tumor properties of EnSCs on EOC cells *in vivo* and *in vitro*. This research provides a potential strategy for EnSC-based anti-cancer therapy against epithelial ovarian cancer.

Epithelial ovarian cancer (EOC) is the most lethal gynecological cancer threatening women worldwide, attributing to the late presentation in most cases, wide abdominal cavity metastasis, high level of recurrence, and frequent formation of chemo-resistance. The standard remedies for EOC mainly include surgery and platinum-based cytotoxic chemotherapy. Although various kinds of treatments, tumor markers and imaging diagnostic techniques have been created for early diagnosis and forestalling recrudesce, invasion or metastasis, 70% of patients will relapse within 18 months^1^. Therefore, there is an urgent need for the development of alternative strategies to improve the outcomes of patients with epithelial ovarian cancer.

Recently, many studies found the intrinsic anti-tumor properties of adult mesenchymal stem cells (MSCs) and showed the potential of MSC-based therapy for treating cancer *in vitro* and in animal models, such as hepatocellular carcinoma^2^, lymphoma^3^, breast cancer^4^, ovarian cancer^4^,^5^, myeloma^6^, and leukemia^7^. Kalamegam *et al*. showed that the conditioned medium and cell lysate of human umbilical cord MSCs possessed inhibitory properties on ovarian cancer cell line (TOV-122D) *in vitro*^4^. Zhu *et al*. reported that rat bone marrow MSCs could inhibit the growth of cisplatin-resistant SK-OV-3 cells in nude mice^5^.

Human endometrial mesenchymal stem cells (EnSCs) derived from menstrual blood are a novel source for adult mesenchymal stem cells^8^. Compared with other adult MSCs, such as adipose-derived MSCs, bone marrow-derived MSCs, umbilical cord-derived MSCs, EnSCs showed more attractive traits. EnSCs can be easily isolated from menstrual blood by noninvasive acquisition. The noncontroversial extraction is also autologous and thus avoiding critical ethical issues^9^. A large number of reports have shown the therapeutic potential of EnSCs in restoring tissue function, such as treating myocardial infarction^10^, ameliorating stroke^11^, and improving chemotherapy-induced premature ovarian failure/insufficiency^12^. However, whether EnSCs endow intrinsic anti-tumor properties on cancer including epithelial ovarian cancer has never been reported before.

In the current study, we established a tumor-bearing mouse model by subcutaneous co-injection of EOC cell line SK-OV-3 and EnSCs. Furthermore, we used EnSCs conditioned medium (EnSC-CM) or transwell system to evaluate their effects on the biological behavior of EOC cells *in vitro*, including cell viability, cell cycle progression, regulation of mitochondrial membrane potential (MMP), and apoptosis of EOC cells. Finally, we elucidated whether EnSCs exerted anti-tumor properties through affecting AKT/Forkhead box O3a (FoxO3a) axis in EOC cells.

## Results

### EnSCs inhibited the growth of EOC cells *in vitro* and *in vivo*

**Figure 1:**
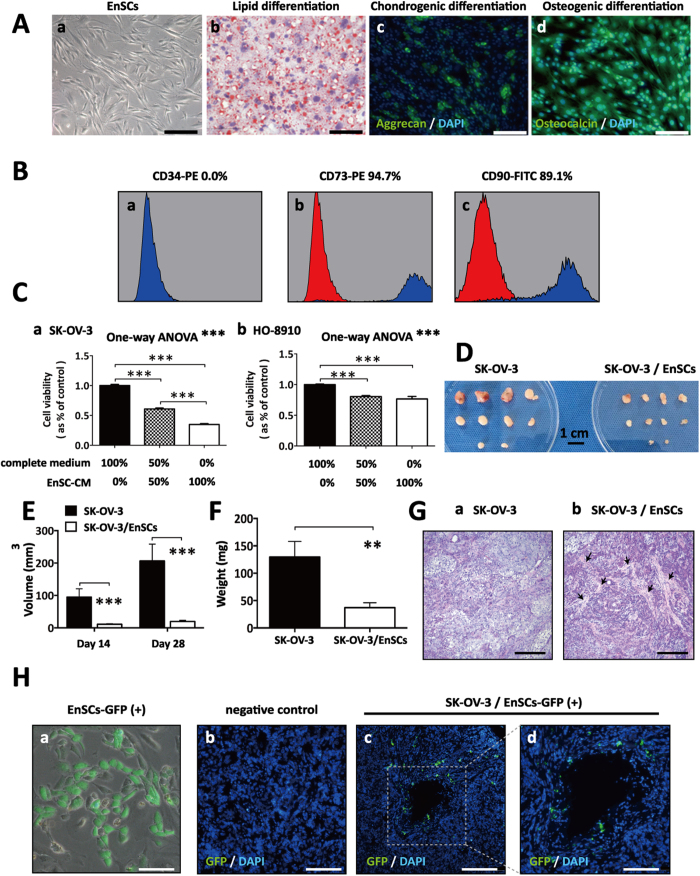
EnSCs inhibited the growth of EOC cells *in vitro* and *in vivo*. **(A**) Representative images showed the mesenchymal morphology (a), lipid differentiation ability (b), chondrogenic differentiation ability (c), and osteogenic differentiation ability (d) of EnSCs (Scale bar = 100 μm). **(B**) Flow cytometry analysis showed that EnSCs were positive for mesenchymal markers, CD73 and CD90, and were negative for marker CD34 expressed in hematopoietic stem cells. **(C**) CCK-8 cell viability assay was used to test the effects of EnSC-CM in different concentrations on the viability of EOC cells at the 48th hour (n = 3; performed in triplicate). Ordinary one-way ANOVA was used for statistic analysis. **(D**) Gross observation of subcutaneous xenografts obtained from SK-OV-3 (2 × 10^6^) group and SK-OV-3/EnSCs (2 × 10^6^: 1 × 10^6^) group (Scale bar = l cm). **(E**) The average tumor volume of SK-OV-3 injected group was significantly bigger than that of SK-OV-3/EnSCs co-injected group day 14 and day 28 (n = 10). **(F**) The average tumor weight of SK-OV-3 injected group was significantly higher than that of SK-OV-3/EnSCs co-injected group at day 28 (n = 10). **(G**) H&E staining was performed on the xenografted tumor sections. (a) showed the nested, diffused and solid growth patterns of SK-OV-3 group. Black arrows in (b) showed the stromal components in xenografts obtained from SK-OV-3/EnSCs group (Scale bar = 200 μm). **(H**) (a) Representative image of GFP-transfected EnSCs (Scale bar = 50 μm). (b–d) IF staining was used to test the existence of EnSCs^GFP+^ in the tumor tissues obtained from SK-OV-3/EnSCs^GFP+^ group (Scale bar of b and c = 200 μm; Scale bar of d = 100 μm). All data were shown as means ± SEM. **p-value < 0.01; ***p-value < 0.001.

EnSCs were morphologically similar to fibroblast-like cells (Fig. 1Aa). These cells are multipotent, as showed by their ability to differentiate into adipocytes, chondroblasts and osteoblasts (Fig. 1Ab–d). Flow cytometry was used to detect the phenotype of EnSCs (at 3rd passage), and results showed that around 90% EnSCs were positive for mesenchymal markers (CD90 and CD 73) and were negative for marker of hematopoietic stem cells (CD34) (Fig. 1B).

In CCK-8 cell viability assay, a significant decrease was observed in the EnSC-CM-treated cancer cells compared to cells treated with complete culture medium, which was dose-dependent (Fig. 1C, n = 3). To test whether the anti-tumor properties of EnSCs are specific or not, we evaluated the effects of EnSC-CM on non-malignant cells (293FT and human umbilical vein endothelial cells/hUVECs) using CCK-8 assay. Furthermore, we collected the conditioned medium from non-malignant cells and assess their effects on EOC cells. We found that EnSC-CM increased the viability of 293FT cells, but decreased that of hUVECs *in vitro* (Supplementary Fig. S1A, n = 3). In addition, we observed that both 293FT and hUVEC-CM exerted slight impact on the viability of EOC cells *in vitro* (Supplementary Fig. S1B, n = 3).

In a gross observation, xenogratfs obtained from SK-OV-3 group presented softer texture, more cystic lesions and hemorrhage sites which were not observed in the SK-OV-3/EnSCs group at the end of the animal experiment (Fig. 1D). We also found that EnSCs significantly decreased the volume and weight of xenografts *in vivo* (Fig. 1F,E, n = 10) at day 28, suggesting the anti-tumor effects of EnSCs in tumor microenvironment. By using hematoxylin and eosin (H&E) staining, we observed that the tumor tissues obtained form the SK-OV-3 group presented nested, diffused and solid growth patterns. In contrast, more stromal components were found in tumor tissues obtained from SK-OV-3/EnSCs group (Fig. 1G).

To confirm the existence of EnSCs in xenografted tumor tissues after implantation with SK-OV-3 cells, we labeled EnSCs with green fluorescent protein (GFP) beforehand (Fig. 1H-a). After 28 days of co-injection, immunofluorescence (IF) assay showed the existence of EnSCs^GFP(+)^ in the tumor microenvironment, suggesting that EnSCs played the inhibitory role in local site of the tumor (Fig. 1Hb–d).

### EnSCs inhibited tumor proliferative ability *in vivo* and *in vitro* through the paracrine way

**Figure 2:**
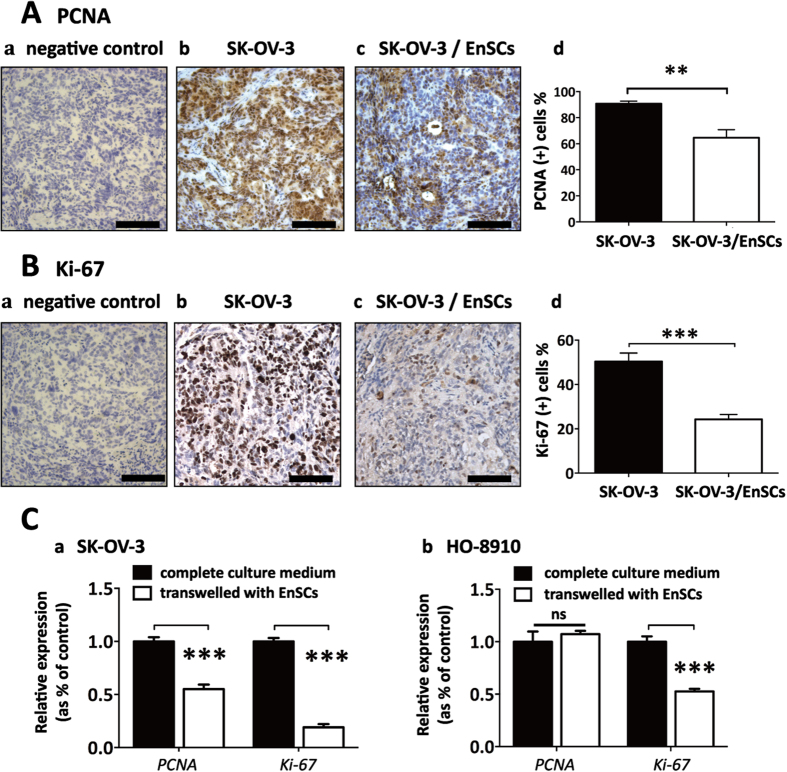
EnSCs inhibited proliferative ability of EOC cells *in vivo* and *in vitro* through the paracrine way. **(A,B**) Proliferative ability of cancer cells were tested by IHC using antibodies against PCNA and Ki-67 in SK-OV-3 and SK-OV-3/EnSCs tumor tissues (n = 5; Scale bar = 100 μm). PCNA and Ki-67 positively-stained cell ratios were measured and results were shown as averages of five randomly selected fields ± SEM. **(C**) Real-time PCR were used to test the effects of EnSCs on the transcription of *PCNA* and *Ki-67* in EOC cells cultured in transwell system for 48 hours (n = 3; performed in triplicate). All data were shown as means ± SEM. ***p-value < 0.001; ns, no statistical significance.

To further confirm whether EnSCs inhibited the proliferation of EOC cells *in vivo*, tumor tissues were stained for proliferating cell nuclear antigen (PCNA) and Ki-67, two indicators for cell proliferative ability. Results showed that the staining of PCNA and Ki-67 were prevailingly localized to the nucleus of malignant cells and the percentages of PCNA (+) and Ki-67 (+) cells were decreased in tissues obtained from SK-OV-3/EnSCs group compared to that obtained from the SK-OV-3 group (Fig. 2A,B, n = 5). In *ex vivo* experiments, transwell system was used to imitate the indirect cell-cell communication between EOC cells and EnSCs. Results from real-time polymerase chain reaction (PCR) showed that EnSCs secretions significantly decreased the transcription of *PCNA* and *Ki-67* in SK-OV-3 cells which were consistent with the *in vivo* observations (Fig. 2Ca, n = 3). However EnSCs only significantly decreased the expression of *Ki-67* in HO-8910 cells (Fig. 2Cb, n = 3).

### EnSCs inhibited cell cycle progression of EOC cells by inducing G0/G1 cell cycle arrest through the paracrine way

**Figure 3:**
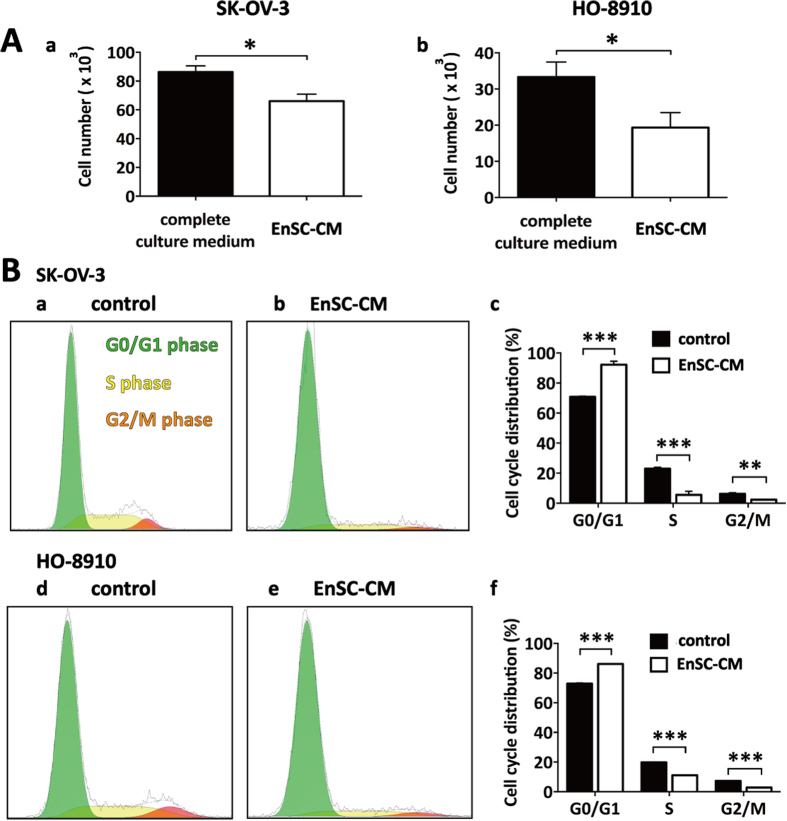
EnSCs inhibited cell cycle progression of EOC cells through inducing G0/G1 cell cycle arrest through the paracrine way. **(A**) The effects of EnSC-CM on the division of EOC cells were tested by cell counting assay. EOC cells were cultured in complete medium or EnSC-CM for 48 hours, then cells were harvested and cell numbers were counted (n = 3; performed in triplicate). **(B**) The effects of EnSCs on cell cycle distribution of EOC cells were tested by flow cytometry using PI staining method (n = 3; performed in triplicate). All data were shown as means ± SEM. *p-value < 0.05; **p-value < 0.01; ***p-value < 0.001.

In cell counting assay, the results showed that EnSC-CM significantly decelerated the division of EOC cells compared to the cells cultured with complete medium (Fig. 3A, n = 3), suggesting a possible role of EnSCs in the regulation of cell cycle progression of EOC cells. We observed that EnSC significantly halted the cancer cells in G0/G1 phase after being treated with EnSC-CM for 48 hours by using flow cytometry (Fig. 3B, n = 3). EnSC-CM also decreased the percentage of cells in both S phase and G2/M phase in comparison to the control group. Moreover, we observed that EnSC-CM alone did not arrest cancer cells in subG1 phase (apoptotic cell peak) (Fig. 3Bb,e, n = 3).

### EnSCs promoted cleavage of caspase 3 *in vivo* and enhanced cisplatin-induced apoptosis in EOC cells through the intrinsic mitochondrial apoptotic pathway

**Figure 4:**
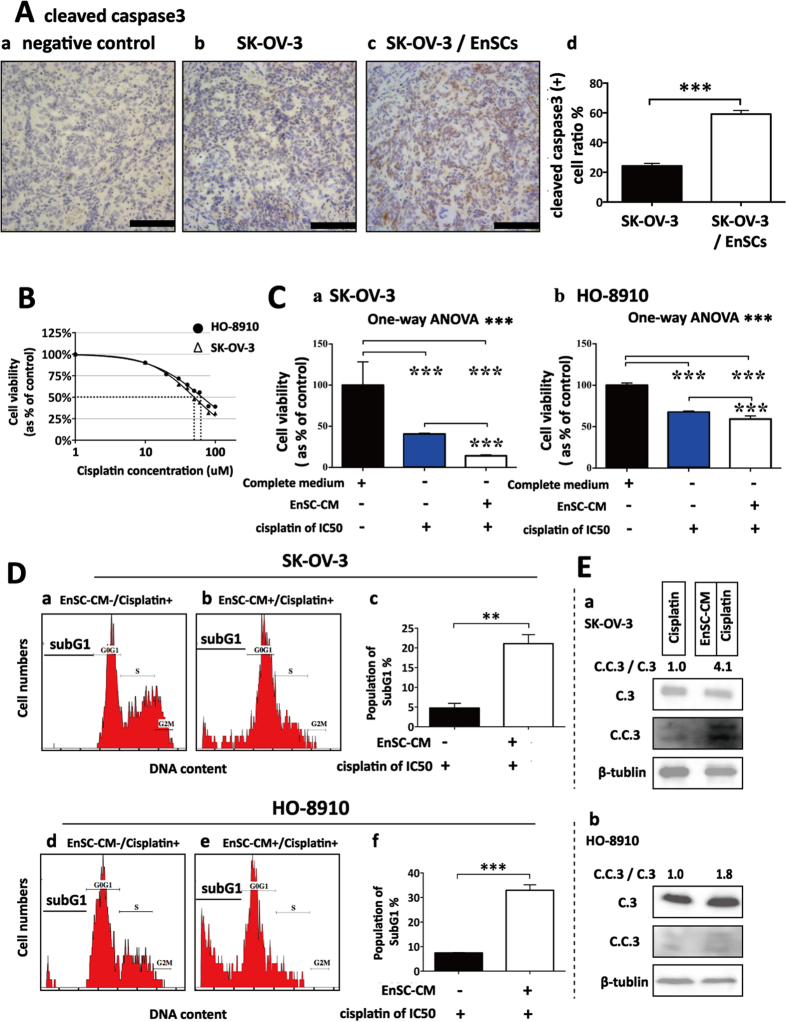
EnSCs promoted apoptosis in EOC cells *in vivo* and enhanced cisplatin-induced apoptosis *in vitro*. **(A**) Apoptosis rate in tumor tissues was tested by IHC using antibody against cleaved caspase 3 (n = 5; Scale bar = 100 μm). Positively-stained cell ratios were measured and results were shown as averages of five randomly selected fields ± SEM. **(B**) The IC50 of cisplatin on SK-OV-3 cells and HO-8910 cells at the 48th hour was tested by CCK-8 assay (n = 3). **(C**) The effects of EnSC-CM on the cisplatin-induced cell death in EOC cells at the 48th hour were tested by using CCK-8 assay (n = 3; performed in triplicate). Ordinary one-way ANOVA was used for statistic analysis. **(D**) The effects of EnSC-CM on cisplatin-induced apoptosis in EOC cells were tested by subG1 assay using flow cytometry (n = 3; performed in triplicate). **(E**) Western blot analysis was performed on cell lysates harvested from EOC cells treated with cisplatin of IC50 with or without EnSC-CM for 48 hours by using antibody against both caspase 3 and cleaved caspase 3 (n = 3; performed in triplicate). The amount of protein loaded was normalized against β-tubulin. All data were shown as means ± SEM. **p-value < 0.01; ***p-value < 0.001.

Next, we investigated whether EnSCs could trigger apoptosis in EOC cells *in vivo* and *in vitro*. Immunohistochemistry (IHC) assay was used to test the level of cleaved caspase 3 in the tumor tissues, which is the key executor of apoptosis in the intrinsic apoptosis pathway^13^. We observed that the percentage of cleaved caspase 3 (+) cells were increased in tumor tissues obtained from SK-OV-3/EnSCs group compared with that obtained from the control group (Fig. 4A, n = 5). To better evaluate the pro-apoptotic effects of EnSCs on EOC cells, we used cisplatin, a widely used chemotherapeutic drug, as an apoptotic stimulus. We assessed whether EnSCs could enhance cisplatin-induced apoptosis in EOC cells. Firstly, the half-inhibitory concentration (IC50) of cisplatin at the 48th hour on SK-OV-3 and HO-8910 cells were tested using CCK-8 assay. Results showed that the IC50 of SK-OV-3 and HO-8910 were 50 μM and 60 μM respectively (Fig. 4B, n = 3). Then we observed that EnSC-CM significantly promoted cisplatin-induced growth inhibition of EOC cells at the 48th hour (Fig. 4C, n = 3). To further evaluate their pro-apoptotic properties, flow cytometry was performed to detect subG1 peak and western blot was performed to detect the expressions of caspase 3 and cleaved caspase 3 in cisplatin-treated EOC cells with or without EnSC-CM. Results showed that the EnSC-CM significantly increased the percentage of cells in subG1 phase and promoted the cleavage of caspase 3 within cisplatin-treated EOC cells compared with the control group (Fig. 4D,E, n = 3).

**Figure 5:**
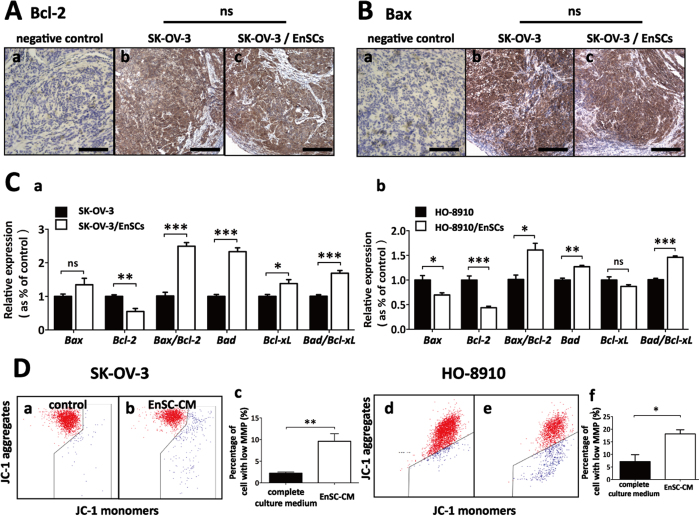
EnSCs impaired regulation of Bcl-2 family and decreased mitochondrial membrane potential (MMP) in EOC cells *in vitro*. **(A,B**) Intrinsic apoptosis pathway in tumor tissues was tested by IHC using antibodies against Bcl-2 and Bax (n = 5; Scale bar = 100 μm). H-score system was used for semi-quantification and no statistic significance (ns) of Bcl-2 and Bax were observed between the two groups. **(C**) Real-time PCR were used to test the effects of EnSCs on the transcription of Bcl-2 family (*Bax, Bcl-2, Bax: Bcl-2* ratio, *Bad, Bcl-x*_*L*_, and *Bad: Bcl-x*_*L*_ ratio) in EOC cells cultured with EnSCs in transwell system for 48 hours (n = 3; performed in triplicate). **(D**) The effects of EnSC-CM on MMP were tested by flow cytometry using mitochondrial probe (JC-1) (n = 3; performed in triplicate). All data were shown as means ± SEM. *p-value < 0.05; **p-value < 0.01; ***p-value < 0.001.

To further assess the impact of EnSCs on regulating the intrinsic apoptotic pathway *in vivo*, IHC assay was used to detect the expressions of pro-apoptotic protein Bax and anti-apoptotic protein Bcl-2 in xenografted tumor tissues. Unexpectedly, we did not observe significant differences in the expression of both Bax and Bcl-2 between SK-OV-3 group and SK-OV-3/EnSCs group (Fig. 5A,B, n = 5). However, we found that EnSCs significantly increased *Bax/Bcl-2* ratio and *Bad/Bcl-x*_*L*_ ratio at the transcriptional level within EOC cells in transwell system at the 48th hour, suggesting the dysfunction of mitochondria (Fig. 5C, n = 3). It is reported that loss of mitochondrial membrane potential (MMP) is a hallmark and an early event of apoptosis, coinciding with caspase activation^14^. To demonstrate the effects of EnSCs on mitochondrial function, mitochondrial probe 5,5′,6,6′-Tetrachloro-1,1′,3,3′-Tetraethylbenzimidazolyl-Carbocyanine iodide (JC-1) was used to stain EnSC-CM-treated cancer cells and then tested by flow cytometry. JC-1 exists as dimer and accumulates as aggregates in the mitochondria, which appears red. But in apoptotic and necrotic cells, JC-1 exists as a monomeric form and stains the cytosol green^15^. In comparison with control, cancer cells exposed to EnSC-CM for 48 hours exhibited a significant decrease in JC-1 aggregates and an increase in the percentage of cancer cells with low MMP (Fig. 5D, n = 3), indicating an impaired function of mitochondria in EOC cells.

### EnSCs inhibited AKT phosphorylation in both paracrine way and direct cell-cell contact way in EOC cells

**Figure 6:**
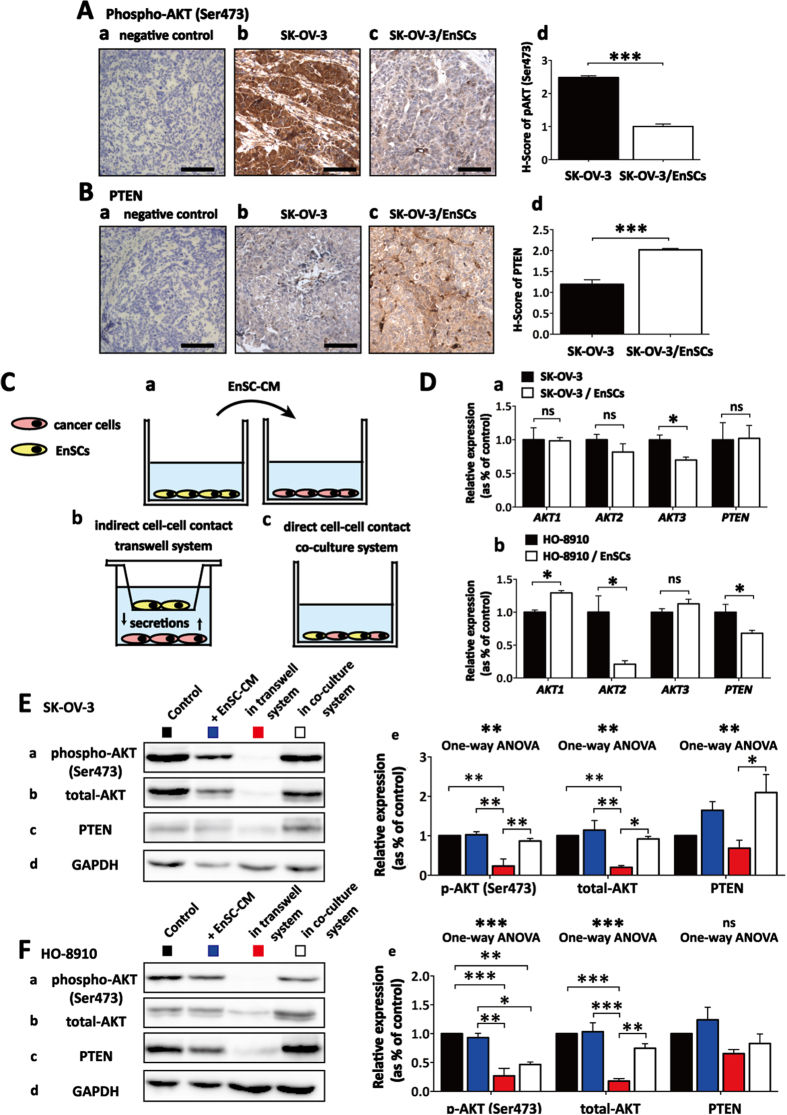
EnSCs inhibited AKT phosphorylation in both paracrine way and direct cell-cell contact way. **(A,B**) The effects of EnSCs on the levels of phospho-AKT (Ser473) and PTEN in the xenografted tumor tissues were tested by IHC using antibodies against phospho-AKT (Ser473) and PTEN (n = 5; Scale bar = 100 μm). H-score system was used for semi-quantification between two groups. **(C**) Diagrammatic drawing of three methods to investigate the inter-communication between EnSCs and EOC cells, including using EnSC-CM (a), transwell system (b) and direct mixed co-culture of EnSCs with EOC cells (c). **(D**) Real-time PCR was used to test the effects of EnSCs on the transcription of *AKT* genes (*AKT1, AKT2, and AKT3*) and *PTEN* in EOC cells cultured with EnSCs in transwell system for 48 hours (n = 3; performed in triplicate). **(E,F**) Western blot was performed to test the effects of EnSCs on the levels of total-AKT, phospho-AKT (Ser473) and PTEN in EOC cells in different culture methods. The amount of protein loaded was normalized against GAPDH (n = 3; performed in triplicate). Ordinary one-way ANOVA was used for statistic analysis. All data were shown as means ± SEM. ns, no statistical significance; *p-value < 0.05; **p-value < 0.01; ***p-value < 0.001.

In xenografted tumor tissues, we found that co-injected EnSCs significantly decreased the level of AKT phosphorylation (serine position 473) and increased the level of phosphatase and tensin homolog deleted on chromosome ten (PTEN), a negative regulator of AKT activation (Fig. 6A,B, n = 5). To distinguish the effects of EnSCs on AKT signaling within EOC cells through either paracrine way or direct cell-cell contact way, we used EnSC-CM, transwell system and direct co-culture system to imitate cell-cell communication respectively (Fig. 6C). The influence of EnSCs on the transcription of *AKTs (AKT1, AKT2* and *AKT3*) and *PTEN* in EOCs was observed. Results from real-time PCR revealed that EnSCs secretions significantly reduced the transcription of *AKT3* in SK-OV-3 cells and reduced the transcription of *AKT2* in HO-8910 cells respectively (Fig. 6D, n = 3). By Western blot, we found that EnSCs significantly decreased the expression levels of phospho-AKT (Ser473) in SK-OV-3 and HO-8910 cells cultured in transwell system, and that in HO-8910 cells cultured in mixed system. There was a decreasing tendency of phospho-AKT (Ser473) level in HO-8910 cells treated with EnSC-CM for 48 hours. EnSCs also significantly decreased the total levels of AKT in both SK-OV-3 and HO-8910 cells in transwell system. Furthermore, we observed that EnSCs increased the expression of PTEN in SK-OV-3 cells treated with EnSC-CM or co-cultured with EnSCs, which was consistent with the *in vivo* results (Fig. 6D,E, n = 3). These data suggested that EnSCs inhibited AKT phosphorylation in both paracrine way and direct cell-cell contact way in EOC cells *in vitro* and *in vivo*.

### EnSCs promoted nuclear translocation of Forkhead box O-class 3a (FoxO3a) and inhibited the pro-angiogenetic ability of EOC cells *in vivo*

**Figure 7:**
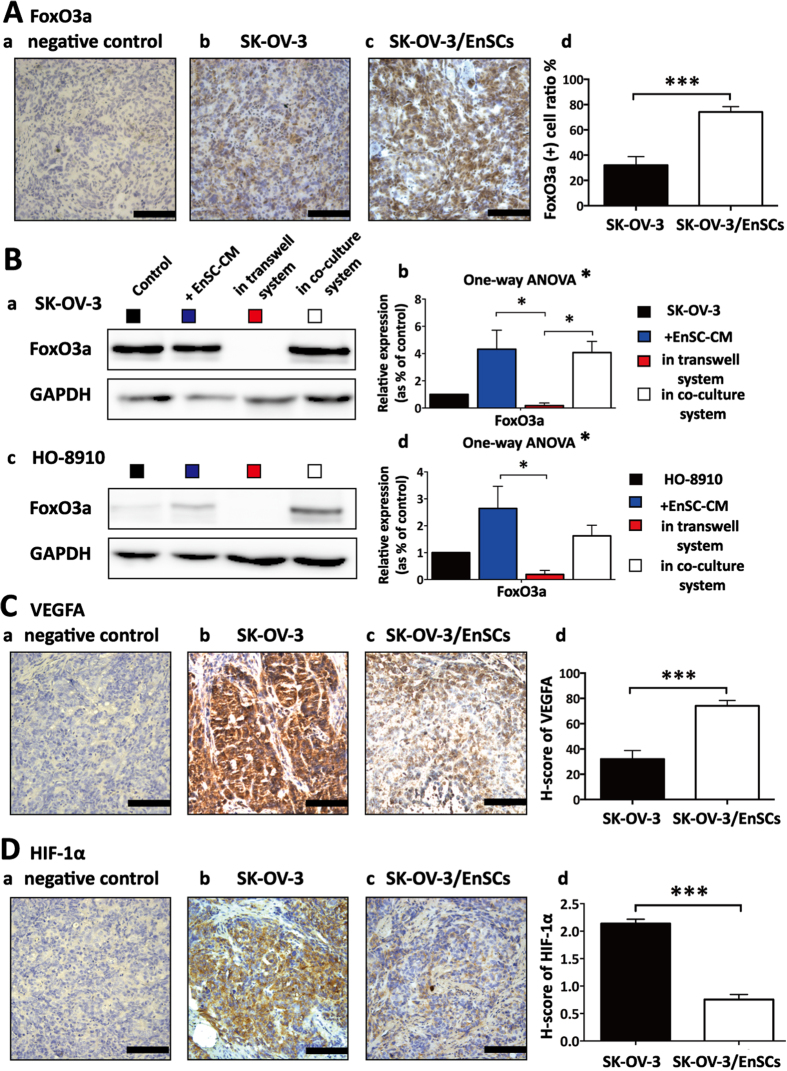
EnSCs promoted nuclear translocation of FoxO3a and inhibited the pro-angiogenetic ability of EOC cells *in vivo*. **(A**) The effects of EnSCs on the levels of FoxO3a in tumor tissues were tested by IHC staining. Positively-stained cell ratios were measured and results were shown as averages of five randomly selected fields ± SEM (n = 5; Scale bar = 100 μm). **(B**) Western blot was performed to test the effects of EnSCs on FoxO3a levels within EOC cells in different culture methods. The amount of protein loaded was normalized against GAPDH (n = 3; performed in triplicate). Ordinary one-way ANOVA was used for statistic analysis. **(C,D)** The effects of EnSCs on the pro-angiogenetic ability of EOC cells *in vivo* were tested by IHC analysis using antibodies against VEGFA and HIF-1α. H-score system was used for semi-quantification between two groups (n = 5; Scale bar = 100 μm). All data were shown as means ± SEM. *p-value < 0.05; **p-value < 0.01; ***p-value < 0.001.

To evaluate the functional status of EnSCs-mediated inhibition of AKT signaling pathway, we further tested the effects of EnSCs on the downstream target of AKT, Forkhead box O-class 3a (FoxO3a). FoxO3a functions as a tumor suppressor, whose transcriptional activity are regulated by many protein kinases such as AKT^16^. Activated AKT inhibits the activity of FoxO proteins by promoting phosphorylation and nuclear exportation, which induces their degradation by the proteasome^16^. In the EnSCs/SK-OV-3 co-injected tumor tissues, IHC analysis showed that the localization of FoxO3a was prevailingly localized to the nucleus of malignant cells. The ratio of FoxO3a positively stained cancer cells was statistically higher than that in the SK-OV-3 injected tumor tissues (Fig. 7A, n = 5). By using EnSC-CM, transwell system and direct co-culture system, we further observed that EnSCs increased the expression levels of FoxO3a in SK-OV-3 cells and HO-8910 cells *in vitro* through both paracrine way and direct cell-cell contact way using western blot (Fig. 7B, n = 3). These results suggested that EnSCs decreased the degradation of FoxO3a and promoted its activity in EOC cells.

Vascular endothelial growth factor (VEGF) and hypoxia-inducible factor-1α (HIF-1α) are important for tumor growth and angiogenesis, which can be regulated by AKT signaling pathway^17^. In the EnSCs/SK-OV-3 xenografted tumor tissues, we observed that the levels of both VEGFA (Fig. 7C, n = 5) and HIF-1α (Fig. 7D, n = 5) was significantly decreased compared with that of SK-OV-3 injected group using IHC analysis, indicating that EnSCs exerted inhibitory effects on the pro-angiogenetic ability of EOC cells *in vivo*.

## Discussion

In this research, we are first to show the anti-tumor properties of EnSCs on epithelial ovarian cancer cells *in vitro* and *in vivo*. To test whether the anti-tumor properties of EnSCs are specific, not owning to the rapid growth of EnSC-induced exhaustion of trophic factors, we tested the impacts of EnSC-CM on non-malignant cells and the effects of conditioned medium collected from non-malignant cells on EOC cells. We found that EnSC-CM exerted dual effects (pro- or anti-proliferation) on the viability of different cells (malignant and non-malignant). In addition, both 293FT and hUVEC-CM exerted weak impact on the viability of EOC cells *in vitro*. Further, we also used transwell system to test the effects of EnSCs secretions on EOC cells, in which EnSC-secreted factors could go through transwell membrane and act on cancer cells. We found that EnSCs also decreased the expressions of *PCNA* and *Ki-67*, indicating EnSCs inhibited the proliferative ability of EOC cells. These data suggested that the EnSC-CM-induced growth inhibition on EOC cells was specific and the inhibitory factors secreted from EnSCs played an important role in this process.

AKT pathway is emerging as a central player in tumorigenesis. Altomare *et al*. reported that activation of AKT (AKT phosphorylation) can be frequently detected in ovarian cancer and can be targeted to disturb ovarian tumor cell growth^18^. Forkhead box O (FoxO) transcription factors, including FoxO1, FoxO3a, FoxO4 and FoxO6, are important family of proteins that exhibit tumor suppression functions by regulating expressions of genes participated in apoptosis, cell cycle progression, angiogenesis, DNA repair, oxidative stress resistance and other cellular functions^19^,^20^,^21^. Levanon *et al*. illustrated that FoxO3a loss is a frequent early event in high grade serous ovarian carcinoma^22^. FoxO3a also shows prognostic value in ovarian cancer patients, whose low expression level is associated with poor prognosis^23^. AKT is reported to promote the degradation of FoxO proteins by the proteasome through increasing their phosphorylation, which will induce the nuclear exportation^16^. In this research, we observed that EnSCs significantly decreased the level of AKT phosphorylation, increased the expression of FoxO3a within EOC cells, and retained more FoxO3a in the nucleus of EOC cells. These results suggested that EnSCs inhibited AKT-mediated degradation of FoxO3a *in vivo* and *in vitro*, which would contribute to the anti-tumor properties of EnSCs on epithelial ovarian cancer.

Errant proliferation is linked to varying cell cycle distribution in ovarian cancer^24^. The cell cycle progression is well-organized by cyclin-dependent kinase (CDK) and CDK inhibitors^25^. FoxO3a activation promotes the transcription of *p21* and *p27*, two cell cycle inhibitory regulators, which subsequently suppress G1 to S cell cycle transition^26^,^27^, which might be associated with the EnSC-induced G0/G1 cell cycle arrest in EOC cells.

It is widely reported that programmed cell death serves as a natural barrier to cancer development and tumor cells evolve various tactics to avoid apoptosis. Active FoxO3 can induce expressions of the pro-apoptotic genes, such as *Bim* and *PUMA*, which function in the intrinsic mitochondrial apoptotic pathway^28^,^29^,^30^. Consistent with these studies, our data showed that EnSC-secreted factors increased *Bax/Bcl-2* ratio and *Bad/Bcl-x*_*L*_ ratio at transcriptional level and reduced mitochondrial membrane potential in EOC cells *in vitro*, indicating the dysregulation of mitochondria and activation of the intrinsic apoptotic pathway. However, our data demonstrated that EnSCs did not affect the levels of Bax and Bcl-2 in tumor tissues obtained form SK-OV-3/EnSCs group compared with that obtained form the SK-OV-3 group. Previous studies reported that even cytotoxic drugs like paclitaxel or cisplatin were unable to up-regulate Bax expression and down-regulate Bcl-2 expression in EOC-bearing mouse models^31^,^32^. Besides, FoxO3a can trigger apoptosis by promoting the expressions of death receptor ligands in the extrinsic apoptotic pathway, such as *Fas ligand*^33^ and *tumor-necrosis-factor-related apoptosis-inducing ligand (TRAIL*)^34^. This mechanism may also participate in the EnSC-induced apoptosis in EOC cells which needs to be elucidated further.

Tumor angiogenesis is an essential event in tumor progression^35^. The vascular endothelial growth factor (VEGF) protein family has a crucial role in regulating vasculogenesis, angiogenesis and lymphangiogenesis^36^. During hypoxia, hypoxia-inducible factor 1 (HIF-1) promotes the expression of *VEGF* to maintain the oxygen homeostasis^37^. Emerling *et al*. reported that FoxO3a negatively regulates the transcriptional activity of HIF-1 through interfering with p300^20^. Karadedou *et al*. demonstrated that FoxO3a represses *VEGF* expression through FOXM1-dependent and -independent mechanisms in breast cancer^19^. Our results showed that EnSCs impaired the pro-angiogenetic ability of ovarian cancer cells *in vivo* through decreasing the expressions of VEGFA and HIF-1α in EOC cells, which might be mediated by EnSC-induced activation of FoxO3a in cancer cells. These data illustrated that EnSC-mediated activation of FoxO3a was important for the innate anti-tumor properties of EnSCs against EOC cells through influencing cellular proliferation, cell cycle progression, apoptosis, mitochondrial function and pro-angiogenetic ability of cancer cells (Supplementary Fig. S2).

MSCs have been shown the tropic properties towards damaged tissues^38^ and tumor microenvironment^39^, which improve the efficiency and target ability of MSC-based therapy. We and other groups have reported the potential application of EnSCs to restore function of injured tissues^9^,^12^,^38^. However, there are some concerns about the potential risk of MSCs. Some researches showed that human MSCs favored the survival and progression of cancer cells through promoting cell proliferation, enhancing invasion/metastasis^40^, and inducing neovascularization^41^. Lalu *et al*. reported that tumorigenicity may occur only in the patients suffering from pre-existing or previous malignancy by conducting a systematic review^42^. In addition, MSCs have the latent risk of malignant transformation and acquiring tumor-supporting ability. Ovarian cancer-derived exosomes enabled MSCs to acquire cancer-associated fibroblast (CAF) characteristics, which contributes to stromal modifications suitable for tumor expansion and stimulates angiogenesis^43^,^44^. In this research, we demonstrated the innate anti-tumor properties of EnSCs against ovarian cancer, however whether ovarian cancer would induce malignant transformation of EnSCs will be further investigated. In addition, MSCs show dual-effects on different kinds of cancer. For instance, bone marrow-derived MSCs exert anti-tumor effects in a model of Kaposi’s sarcoma^45^, but support colon cancer cell growth *in vivo*^46^. These data suggested the necessity of testing the effects of EnSCs on different cancer cells in the future studies.

Genetically engineered MSCs have been acted as promising vehicles of prodrugs^47^, anti-tumor cytokines^48^,^49^,^50^,^51^ or suicide genes^52^ in the anti-tumor therapy against ovarian cancer. Whether EnSCs can be used as recipient cells in gene therapy against ovarian cancer needs to be further elucidated.

Compared with stem cells obtained from other sources, human endometrial mesenchymal stem cells derived from menstrual blood are one of the typical adult mesenchymal stem cells. EnSCs endow many advantages including non-invasive harvest procedure, easy expansion *in vitro*, and no ethical considerations. Therefore, EnSCs are a potential alternative way to treat ovarian cancer and other diseases. However, there are still some challenges like other cell-based therapies^53^,^54^,^55^. Firstly, the fate and long-term efficiency of transplanted EnSCs are still unclear. Secondly, whether the age and healthy conditions will influence the anti-tumor effects of EnSCs need to be confirmed^56^. Thirdly, strict quality control of isolation, expansion and characterization of EnSCs and long-term supervision system should be well-established. Finally, some technical points of EnSC-based therapy need to be explored in depth, such as the delivery routes, correct time, optimal dose, and so on.

In summary, EnSCs exert intrinsic anti-tumor properties against epithelial ovarian cancer *in vitro* and *in vivo*. This research provides an alternative way of EnSC-based anti-tumor therapy against epithelial ovarian cancer. It also illustrates the safety of using non-engineered EnSCs (autologous or heterologous) in treating ovarian diseases, such as premature ovarian failure/insufficiency induced by cancer-related chemotherapy. More basic researches and preclinical studies are needed to further evaluate the safety and efficiency of EnSC-based therapies.

## Materials and Methods

### Cell culture and collection of condition medium

The human EnSCs were isolated from menstrual blood of a 40-year-old Chinese woman according to the protocol previously reported^9^,^38^ with approval from the institutional ethics committee and written informed consent was obtained. EnSCs were cultured in Chang Medium (S-Evans Biosciences, Hangzhou, China) with 5% CO_2_ at 37 °C and were passaged every 4–6 days. The 3rd-4th passage of EnSCs were used for experiments until they reached 80–90% confluence. Human cells project was approved by the Ethics Committee of the International Peace Maternity and Child Health Hospital, Shanghai Jiaotong University, Shanghai, China. Methods were carried out in accordance with the approved guidelines.

The ovarian cancer cell lines (SK-OV-3 cells and HO-8910 cells) and 293FT were obtained from Shanghai Cell Bank of Chinese Academy of Sciences (Shanghai, China) and cultured in DMEM/High glucose medium (Life technologies, USA) supplemented with 10% fetal bovine serum (FBS, Life technologies, USA), 100 U/mL streptomycin (Life technologies) and 100 U/mL penicillin (Life technologies), and incubated with 5% CO_2_ at 37 °C. The human umbilical vein endothelial cell line (hUVECs) is kindly provided by Dr. Fuju Tian (International Peace Maternity and Child Health Hospital, School of Medicine, Shanghai Jiao Tong University, Shanghai, China), which is cultured in the DMEM/F12 supplied with 10% FBS, 100 U/ml penicillin and 100 U/ml streptomycin.

5 × 10^6^ cells of EnSCs, 293FT, hUVECs were seeded in 100-mm diameter dishes respectively (Corning, USA) with complete culture medium for 72 hours. The EnSC-CM, 293FT-CM and hUVEC-CM were then collected, 0.22 mm filtered, and used in subsequent experiments.

### Differentiation assay of EnSCs

The 3rd passage of EnSCs were cultured and treated with human mesenchymal stem cell functional identification kit (R&D System, Minneapolis, Minnesota, USA) according to the manufacturer’s instruction. Cells were then fixed in 4% polyformaldehyde (PFA). For lipid differentiation, cells were stained with Oil Red O for 30 min. For chondrogenic differentiation, cells were stained with antibody against aggrecan. For osteogenic differentiation, cells were stained with antibody against osteocalcin. After being incubated with primary antibodies, cells were incubated with secondary antibody conjugated with Alexa Fluor^®^ 488 (1:200, Thermo Fisher Scientific). Then cells were counterstained with DAPI (Thermo Fisher Scientific) and examined under the fluorescence microscope (Leica, Germany).

### Flow cytometry

#### Characterization of EnSCs

To characterize EnSCs, cells were harvested and stained with labeled primary antibodies according to the technical data sheet as follows: CD34-PE (BioLegend, USA), CD73-PE (BioLegend) and CD90-FITC (BioLegend) for 30 min at 4 °C. Then cells were washed by cold phosphate buffer solution (PBS) and analyzed by a Cytomics™ FC500 flow cytometer (Beckman Coulter, USA). Data were analyzed by Beckman Coulter CXP software.

#### Cell cycle proportion analysis

To assess the effects of EnSCs on cell cycle progression of EOC cells, cancer cells were cultured with or without EnSC-CM. After 48 hours, cancer cells were harvested and stained with propidium iodide (PI). To assess the effects of EnSCs on the cisplatin-induced apoptosis, cisplatin-treated EOC cells were cultured with or without EnSC-CM. After 48 hours, cancer cells were harvested and stained with PI. Protocol of PI staining was described previously^57^.

#### Mitochondrial membrane potential (MMP) analysis

To assess the effects of EnSCs on MMP of EOC cells, EnSC-CM-treated cancer cells or cells cultured in complete medium were harvested and resuspended in serum-free medium. Each sample was added 5 μl of 1X JC-1 dye (Yeasen) for 20 min at 37 °C. The cells were then analyzed by a Cytomics™ FC500 flow cytometer (Beckman). Data were analyzed by Beckman Coulter CXP software.

#### CCK-8 assay

Cell viability was detected by cell counting kit-8 (CCK8, Dojindo, Japan) according to the manufacturer’s instructions. Cancer cells (SK-OV-3 and HO-8910) were harvested and 7000 cells per well were seeded in 96-well plate with complete culture medium or EnSC-CM or 293FTCM or hUVEC-CM or the mixture of complete culture medium and conditioned medium at 1:1 ratio for 48 hours. Non-malignant cells (293FT and hUVECs) were harvested and 7000 cells per well were seeded in 96-well plate with complete culture medium or EnSC-CM for 48 hours. To test the IC50 of cisplatin on SK-OV-3 and HO-8910 cells at the 48th hour, 7000 cells per well were seeded in 96-well plates and treated with the indicated concentrations of cisplatin. After 48 hours, cell viability was measured. To test the effects of EnSC-CM on cisplatin-induced cell death, cancer cells were seeded in 96-well plates (7000 cells per well) in the presence or absence of EnSC-CM and treated with cisplatin of IC50. After 48 hours, cell viability was measured.

#### *In vivo* tumor models

Twelve female BALB/c nude mice (4 weeks old) were obtained from Shanghai Jiao Tong University School of Medicine (Shanghai, China). All experimental protocols were approved by the Ethics Committee of the School of Medicine of Shanghai Jiao Tong University, which were in accordance with the approved guidelines set by the Institutional Animal Care and Use Committee. The mice were housed under a laminar flow hood in an isolated room and were maintained under pathogen-free conditions. Five mice per group were subcutaneously injected with 2 × 10^6^ SK-OV-3 or SK-OV-3/EnSCs (2 × 10^6^: 1 × 10^6^) at both right and left scapular regions. Tumor volume was measured every two weeks and tumor weight was measured at the endpoint of the experiment. Animals were sacrificed by cervical dislocation under anesthetic status after 28 days. Tumor volume was calculated using the formula: tumor volume (mm^3^) = 0.52 × (width [mm^2^]) × (length [mm])^58^. To localize the EnSCs in the tumor microenvironment, we transfected EnSCs with GFP by lentivirus which was kindly provided by the Prof. Lijian Hui (Chinese Academic of Sciences, Shanghai, China). Two mice were used to establish SK-OV-3/EnSCs^GFP^ (2 × 10^6^: 1 × 10^6^) xenografts and were sacrificed after 28 days.

### Morphological analysis of xenografts

#### Hematoxylin and Eosin (H&E) staining

Xenografts were fixed with 4% PFA, dehydrated by a graded ethanol series, immersed in xylene, and embedded in paraffin. Tissue sections were then cut into 5 μm-thick, deparaffinized and stained with hematoxylin and eosin (H&E).

#### immunofluorescence (IF) staining and immunohistochemistry (IHC) staining

Tissue sections were deparaffinized and dehydrated. The slides were then incubated in a 3% H_2_O_2_ solution to block the endogenous peroxidase (IF staining can skip this procedure), followed by rinsing in PBS. To retrieve the antigenicity, sections were then treated with heated antigen retrieval solution containing ethylene diamine tetraacetic acid (EDTA; Wuhan Goodbio technology, Wuhan, China) or improved citrate antigen retrieval solution (Beyotime, China) according to the instructions of specific primary antibodies. After being incubated with 5% bull serum albumin for 30 min to block the nonspecific anti-body binding sites, the samples were then incubated with the following primary antibodies at 4 °C overnight: PCNA (1:6000 dilution; CST, USA), Ki-67 (1:200 dilution; Arigo biolaboratories, Taiwan, China), Bcl-2 (1:100 dilution; Abcam, England), Bax (1:100 dilution; Abcam), cleaved caspase 3 (Asp175) (1:150 dilution; CST), phospho-AKT (Ser473) (1:50 dilution; CST), PTEN (1:125 dilution; CST), FoxO3a (1:800 dilution; CST), VEGFA (1:25 dilution; Abcam), HIF-1α (1:200 dilution; Wanleibio, China) and GFP (1:100 dilution; CST). For IHC analysis, horseradish peroxidase (HRP)-labelled anti mouse/rabbit second antibodies and diaminobenzidine were used according to the manufacturer’s instructions (Wuhan Goodbio technology). Slides were counterstained with hematoxylin, differentiated with 0.1% hydrochloric acid alcohol, and washed by water. They were then dehydrated through a graded ethanol series, immersed in xylene and mounted with permount^TM^ mounting medium. For IF analysis for GFP, tissues were stained with secondary antibody conjugated with Alexa Fluor^®^ 488 (1:200, Thermo Fisher Scientific). Then tissues were counterstained with DAPI (Thermo Fisher Scientific) and examined under the fluorescence microscope (Leica, Germany).

For the semi-quantitative evaluation of the levels of Bcl-2, Bax, p-AKT, PTEN, VEGFA and HIF-1α, we used a scoring method described by Kenneth S. McCarty, Jr^59^ which classified the immunostaining intensity into four categories: not present (0), weak but detectable above control (1+), distinct (2+), and very strong (3+) and the H-Score was calculated as 1 × (% cells 1+) + 2 × (% cells 2+) + 3 × (% cells 3+). The evaluation of PCNA, Ki-67, cleaved caspase 3 and FoxO3a were measured by the percentage of cells with positive signals in the nucleus. H-Scores or positive rates between co-injected group and control group were measured and analyzed by two-tail Student’s t-test.

#### Indirect culture system and direct co-culture system of EOC cells and EnSCs

To imitate indirect cell-cell communication between EnSCs and EOC cells, transwell system or EnSCs conditioned medium (EnSC-CM) were used in *ex vivo* experiments. 1 × 10^5^ EnSCs were seeded on the transwell insert with a 0.4 μm pore size membrane (Corning) and 2 × 10^5^ EOC cells were seeded in a 6-well culture plate at the lower compartment. In direct co-culture system, 2 × 10^5^ EOC cells were co-seeded with 1 × 10^5^ EnSCs in a 6-well culture plate. All groups were cultured with Chang Medium and incubated with 5% CO_2_ at 37 °C. After 48 hours, cancer cells were collected for subsequent experiments.

#### RNA extraction and quantitative real time polymerase chain reaction (PCR)

Total RNA was extracted from EOC cells by Trizol (Life technologies) according to the manufacturer’s instructions. Real-time PCR were performed by using the SYBR Green Real-time PCR Master Mix (Takara, Japan). PCR primers were designed according to the cDNA sequences in the NCBI database. Primer sequences used for real-time PCR are listed in Supplementary Table S1. Cycling conditions for the PCR machine were set as follows: 95 °C 5 s, 60 °C 30 s and 72 °C 30 s for 40 cycles. All reactions were performed in a 10 μL volume. Gene expression levels were evaluated by using the delta-delta CT method and were standardized to the levels of 18 s RNA amplification.

#### Western blot analysis

For Western blot analysis, total protein lysate from ovarian cancer cells was prepared, separated on 10% sodium dodecyl sulfate polyacrylamide gel electrophoresis and transferred to a polyvinyldifluoridine membrane (Millipore, USA). Membranes were blocked with 10% non-fat milk in Tris-HCL buffer solution containing 0.1% Tween-20 (TBST) and were separately incubated in the following primary antibodies at 4 °C overnight: beta-tublin (1:10000 dilution; Yeasen, Shanghai, China), GAPDH (1:5000 dilution; Yeasen), caspase 3/cleaved caspase 3 (1:1000 dilution; CST), phospho-AKT (Ser473) (1:1000 dilution; CST), AKT (pan) (1:1000 dilution; CST), PTEN (1:1000 dilution; CST), FoxO3a (1:1000 dilution; CST). After washing with TBST, membranes were incubated with HRP-conjugated anti-rabbit or anti-mouse IgG (1:5000; Abcam). Visualization of blots was performed using a standard protocol for electrochemiluminescence (New Cell & Molecular Biotech Co, Jiangsu, China). Levels of beta-tublin or level of GAPDH were used as internal standards.

### Statistical analysis

Results from three independent experiments are reported as the mean ± SEM. Two-tail Student’s t-test or ordinary One-way analysis of variance (ANOVA) with Tukey’s multiple comparisons test was used to evaluate statistical differences via GraphPad Prism version 6 (GraphPad Software). Differences were considered statistically significant when p-value was <0.05.

## Additional Information

**How to cite this article**: Bu, S. *et al*. Human endometrial mesenchymal stem cells exhibit intrinsic anti-tumor properties on human epithelial ovarian cancer cells. *Sci. Rep.* **6**, 37019; doi: 10.1038/srep37019 (2016).

**Publisher’s note**: Springer Nature remains neutral with regard to jurisdictional claims in published maps and institutional affiliations.

## Supplementary Material

Supplementary Information
