# Supplementary material for: Human endometrial mesenchymal stem cells exhibit intrinsic anti-tumor properties on human epithelial ovarian cancer cells
Source: Sci Rep. 2016 Nov 15;6:37019. doi: 10.1038/srep37019 (PMC5109482; doi:10.1038/srep37019)
Supplement: Supplementary Information [file srep37019-s1.pdf]

Human endometrial mesenchymal stem cells exhibit intrinsic anti-tumor properties on human epithelial ovarian cancer cells.

Shixia Bu[1], Qian Wang[1], Qiuwan Zhang[1], Junyan Sun[1], Biwei He[1], Charlie Xiang[2], Zhiwei Liu[1*] and Dongmei Lai[1*]

[1]The International Peace Maternity and Child Health Hospital, School of Medicine, Shanghai Jiaotong University, Shanghai 200030, China
[2]State Key Laboratory for Diagnosis and Treatment of Infectious Diseases, the First Affiliated Hospital, School of Medicine, Zhejiang University, Hangzhou 31000

Correspondence information: Professor Dongmei Lai & Zhiwei Liu, The International Peace Maternity and Child Health Hospital, School of Medicine, Shanghai Jiaotong University, Shanghai 200030, China. Telephone: 021-64070434; Fax: 86-21-64074642; e-mail: laidongmei@hotmail.com and liuzhiwei@hotmail.com.

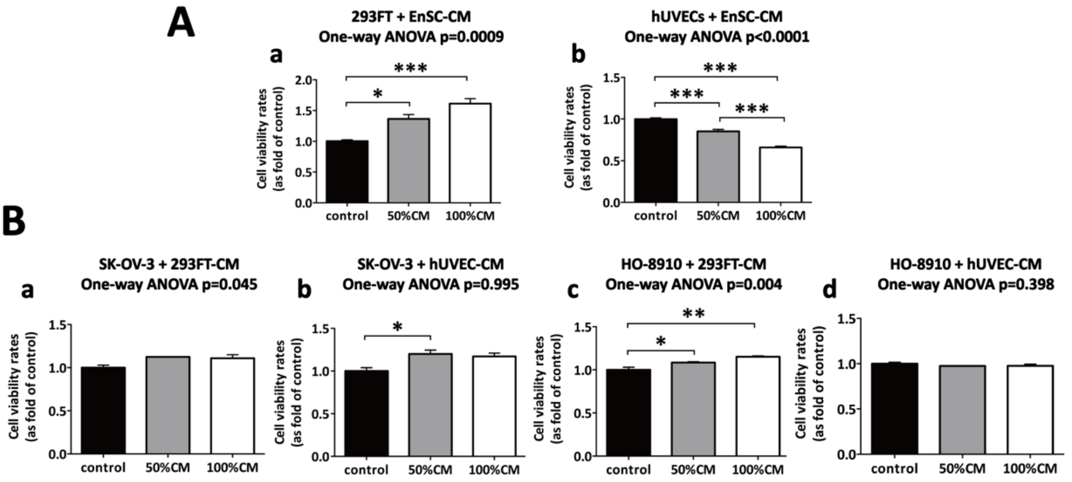

Supplementary Figure S1. The anti-tumor properties of EnSCs against EOC cells through the paracrine pathway is specific. **(A):** CCK-8 cell viability assay was used to test the effects of EnSC-CM on the viability of non-malignant cells (293FT and hUVECs) at the 48th hour (n=3; performed in triplicate). **(B):** CCK-8 cell viability assay was used to test the effects of 293FT-CM and hUVEC-CM on the viability of EOC cells at the 48th hour (n=3; performed in triplicate). All data were shown as means ± SEM. Ordinary one-way ANOVA was used for statistic analysis. *p-value<0.05; **p-value<0.01; ***p-value<0.001.

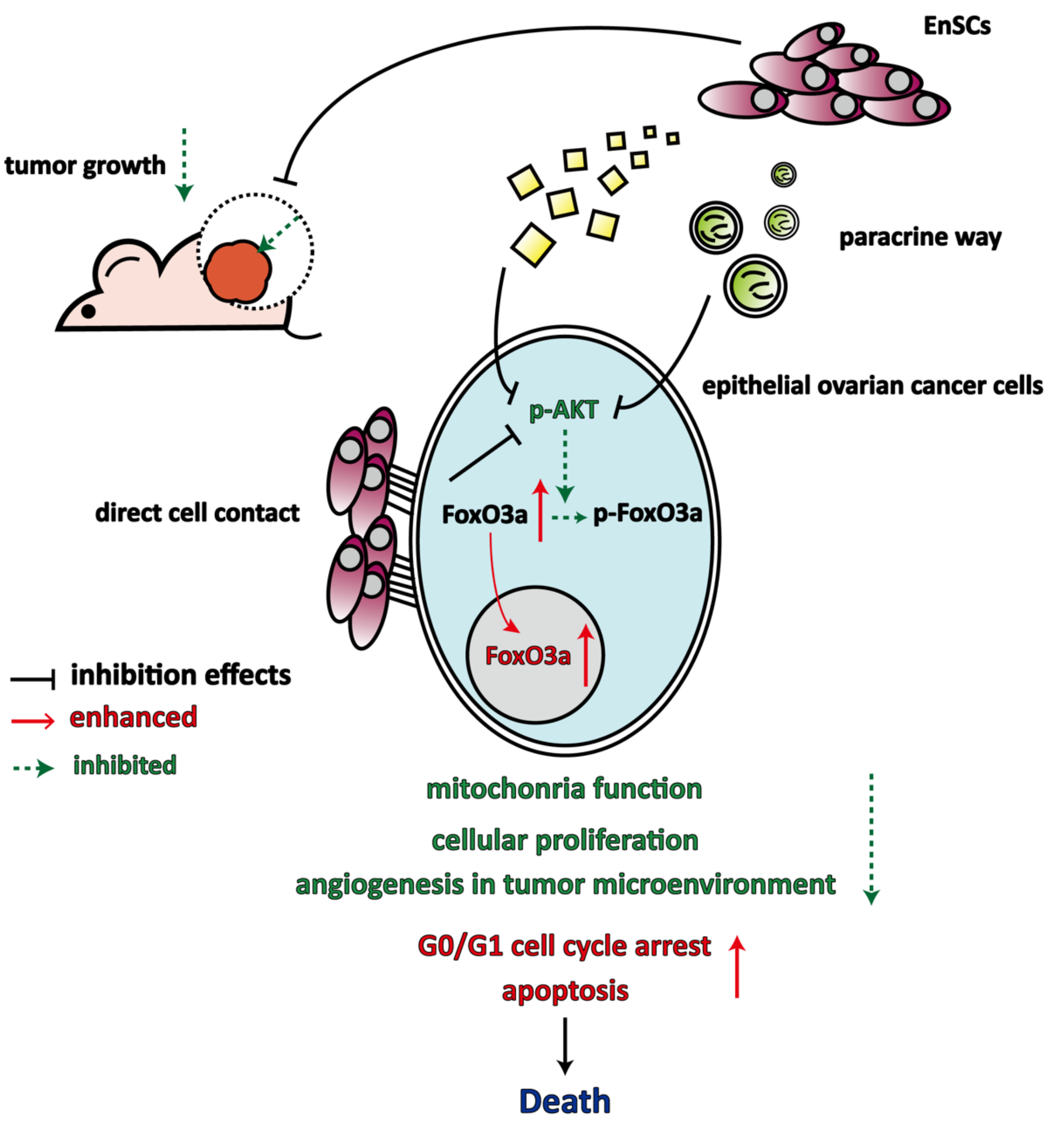

Supplementary Figure S2. A schematic diagram showed that EnSCs exhibited anti-tumor effects on EOC cells through inhibiting AKT phosphorylation and promoting nucleus translocation of FoxO3a in EOC cells in vivo and in vitro through both paracrine way and direct cell-cell contact way. The EnSC-induced transcriptional activity of FoxO3a induces mitochondria dysfunction, cellular growth inhibition, G0/G1 cell cycle arrest, apoptosis and inhibition of pro-angiogenetic ability of EOC cells in vivo and in vitro.

Supplementary Table S1

Primers for real-time PCR analysis of human genes.

Gene	Primer sequence	Product size (bp)
**18s RNA**	Forward: 5′-CGTTGATTAAGTCCCTGCCCTT-3′<br>Reverse: 5′-TCAAGTTCGACCGTCTTCTCAG-3′	137
**PCNA**	Forward: 5′-TTCCTGTGCAAAAGACGGAG-3′<br>Reverse: 5′-TCACCGTTGAAGAGAGTGGA-3′	197
**Ki-67**	Forward: 5′-GAGGCAAATCATCCGAACCC-3′<br>Reverse: 5′-TTATTTTGGCGTCTGGAGCG-3′	181
**Bcl-2**	Forward: 5′-ATGTGTGTGGAGAGCGTCAACC-3′<br>Reverse: 5′-TGAGCAGAGTCTTCAGAGACAGCC-3′	196
**Bax**	Forward: 5′-AAGAAGCTGAGCGAGTGTCT-3′<br>Reverse: 5′-TGGCAAAGTAGAAAAGGGCG-3′	184
**Bcl-x_L**	Forward: 5′-AGTTCAGCACCACCCTAGTC-3′<br>Reverse: 5′-GCTGTTCCTGATAGCTCCCT-3′	212
**Bad**	Forward: 5′-GAGTGACGAGTTTGTGGACT-3′<br>Reverse: 5′-TTTCGGGATGTGGAGCGAA-3′	178
**AKT1**	Forward: 5′-TCGGCAAGGTGATCCTGGTGAA-3′<br>Reverse: 5′-CGGTCGTGGGTCTGGAAAGAGT-3′	184
**AKT2**	Forward: 5′-AGGAGATGGAAGTGGCGGTCAG-3′<br>Reverse: 5′-GGTCGTGGGTCTGGAAGGCATA-3′	270
**AKT3**	Forward: 5′-GCAAAGGATGAAGTGGCACACA-3′<br>Reverse: 5′-TCCTCAGAGAACACCCGCTCTC-3′	182

**PTEN**	Forward: 5′-GAGGCAGCCGTTCGGAGGATTA-3′<br>Reverse: 5′-ATGGCTCTGGACTTGGCGGTAG-3′	196
